# Angiogenic properties of dental pulp stem cells conditioned medium on endothelial cells *in vitro* and in rodent orthotopic dental pulp regeneration

**DOI:** 10.1016/j.heliyon.2019.e01560

**Published:** 2019-04-28

**Authors:** Sueli Patricia Harumi Miyagi de Cara, Clarice Silvia Taemi Origassa, Fernando de Sá Silva, Maria Stella N.A. Moreira, Danilo Candido de Almeida, Ana Clara Fagundes Pedroni, Giovanna Lopes Carvalho, Diego Pulzatto Cury, Niels Olsen Saraiva Câmara, Márcia Martins Marques

**Affiliations:** a School of Dentistry, Centro Universitário das Faculdades Metropolitanas Unidas (FMU), São Paulo, SP, Brazil; b Departamento de Medicina, Divisão de Nefrologia, Universidade Federal de São Paulo, São Paulo, SP, Brazil; c Institute of Life Sciences, Universidade Federal de Juiz de Fora (UFJF), Governador Valadares, MG, Brazil; d School of Dentistry, Universidade Ibirapuera (UNIB), Sao Paulo, SP, Brazil; e Department of Restorative Dentistry and Endodontics, School of Dentistry, University of Sao Paulo (USP), Sao Paulo, SP, Brazil; f School of Medicine, UNINOVE University, São Paulo, São Paulo, Brazil

**Keywords:** Bioengineering, Cell biology

## Abstract

**Objectives:**

To evaluate the effect of SHED-CM on the proliferation, differentiation, migration ability, cell death, gene expression and production of VEGF of HUVEC *in vitro* and in a rodent orthotopic dental pulp regeneration.

**Methods:**

Three culture media [M199, DMEM/Ham's F12 and DMEM/Ham's F12 conditioned by SHEDs] were used as experimental groups. SHED-CM was prepared maintaining confluent cells in culture without serum for 3 days. The proliferation and cell death marker of HUVECs were assessed using flow cytometry. The capacity of formation of vascular-like structures was analyzed in cells grown over Matrigel^®^ in hypoxic condition. HUVECs migration was followed using the scratch test. VEGF-A expression in HUVECs was assessed using real time RT-qPCR; and VEGF synthesis with ELISA test. SHED-CM was also applied in rodent ortotopic model of dental pulp regeneration in rats. The formed tissue was submitted to histological and immunohistochemical analyses.

**Results:**

SHED-CM promoted significantly lower expression of 7AAD in HUVECs; whereas the expression of the Ki67 was similar in all groups. The vascular-like structures were observed in all groups. Migration of SHED-CM group was faster than DMEM/Ham's F12. SHED-CM induced similar expression of VEGF-A than M199, and higher than DMEM/Ham's F12. SHED-CM induced significantly higher VEGF synthesis than other media. SHED-CM induced formation of a vascularized connective tissue inside the root canal.

**Conclusion:**

The study showed that SHEDs release angiogenic and cytoprotective factors, which are of great importance for tissue engineering.

**Clinical significance:**

SHED-CM could be an option to the use of stem cells in tissue engineering.

## 1 Introduction

Tissue engineering aims to restore lost tissues and represents an important research area in the modern Dentistry. The total replacement of pulp tissue lost due traumatic reasons, infection, inflammation or necrosis would be of great relevance in Regenerative Dentistry. Currently, this technology involves three basic elements: stem cells and scaffolds modulated by adhesion, proliferation and differentiation factors [1] and an efficient blood/nutrients supply.

Adult stem cells (ASCs) are undifferentiated cells that can be isolated from adult tissues (or already formed). These cells have self-renewal and multipotent differentiation capacity and have less teratogenic potential compared to embryonic stem cells [2, 3]. In the oral cavity, the ASCs can be isolated from various healthy or inflamed tissues such as: pulp of permanent teeth [4]; pulp of exfoliated deciduous teeth (SHEDs) [5]; periodontal ligament [6]; apical papilla (SCAPs) [7]; dental follicle [8]; human periapical cyst [9] and gingiva [10].

Dental pulp is an important source of ASCs because: a) these cells are easy to be obtained; b) autologous cells may be isolated and amplified from deciduous or permanent teeth; c) they differentiate faster into odontoblasts than other cell sources and produce dentin; d) these cells have immunomodulatory and anti-inflammatory capacity when used in allotransplantation experiments [11, 12, 13], generally at low passages [14]. In addition, cells of primary teeth (SHEDs) and those from dental papilla showed to be more proliferative then DPSCs [15, 16]. Studies have shown also the angiogenic potential of other dental-derived stem cells *in vitro* and *in vivo* [17, 18].

Culture media collected from cells in culture contain released small molecules metabolites and growth factors [19] and are known as conditioned medium. To substitute the cells in tissue engineering, the conditioned medium, containing high concentration of proactive factors, is used as a strategy aiming to decrease the incidence of rejection after the transplants [11]. Conditioned medium (CM) induced by confluent stem cells in culture during a certain period, without the influence of external factors, allows the accumulation of factors released only by these cells. Recent studies have shown that CM by endothelial progenitor cells [20, 21] and SCAPs [22] in hypoxia condition stimulates the angiogenesis process by releasing growth factors resulting in cell proliferation. In addition, hypoxia also has the ability to promote vascular remodeling as observed in pulmonary ischemia cases [23].

The replacement of any oral tissue such as soft tissue, bone, teeth or the periodontium is directly related to an appropriate local vascularization. In Dentistry, the endodontic treatment is a factor that can weaken the remaining dental structure due to the pulp tissue removal. Even after the tooth rehabilitation, the absence of the nutrition and hydration from the pulp, the tooth may become dried and fragile. In these cases, it would be interesting to replace the pulp tissue through the regenerative endodontic procedures, which are dependent of neovascularization. Thus, with the hypothesis that SHED-CM would contain factors capable to stimulate the neovascularization process; in this study we have evaluated the influence of the SHED-CM on human umbilical vein endothelial cells (HUVECs) *in vitro*, for proliferation, vessels-like structures formation ability, migration, cell death, gene expression and production of VEGF. Moreover, an *in vivo* rodent ortotopic model of regenerative endodontics was used to analyze the effects of the SHED-CM in the dental pulp regeneration.

## 2 Materials and methods

For this research, SHEDs from the Basic Research Laboratory Cell Bank (School of Dentistry, University of Sao Paulo - FOUSP) and HUVEC from Federal University of São Paulo (UNIFESP) were obtained following the protocols approved by the Local Ethics Committee in Research (Protocols nº #736.005/2014 and #767.778/2014, respectively). The experiment using the rodent ortotopic model of dental pulp was approved in the following document (Protocol nº #1112/2011).

### 2.1 Cell culture

Two cell types were used in this investigation, as follows: stem cells from human exfoliated deciduous teeth [SHEDs; PDH3 cells (a mixed cell population enriched by stem cells)] and the human umbilical vein endothelial cells (HUVECs). SHEDs were characterized, expanded and stored in liquid N_2_ cryoprotected with DMSO (Sigma Chemical, St Louis, MO, USA).

Analysis of the immunoprofile of the surface molecules using flow cytometry was used for assessing the nature of the PDH3 cells. Aliquots of the PDH3 cells (1 × 10^5^ cells) were washed and resuspended in phosphate buffered saline (PBS) containing the conjugated primary monoclonal antibodies (1:200; BD Biosciences, San Diego, CA, US) that are specific to mesenchymal stem cells (MSCs)-associated (CD44 and CD146) and non-associated (CD14 and CD45) markers. A total of 50.000 events were analyzed by flow cytometer (FACS Canto, BD Bioscences) using the 9.6.2 FlowJo Software Version (Tree Star). For the experiments the cells were thawed and cultivated with DMEM/Ham's F12 (clonogenic medium; 1:1; Invitrogen, Carlsbad, CA, US) supplemented with 15% fetal bovine serum (Hyclone, Logan, UT, US), 2 mM L-glutamine (Invitrogen), 100 μg/mL streptomycin (Invitrogen), 100 U/mL penicillin (Invitrogen) and 2 mM non-essential amino acids (Invitrogen) and kept in a humid atmosphere at 37 °C containing 5% CO_2_. The culture medium was changed every 48 or 72 hours accordingly to the cell metabolism. PDH3 were used at passages P3 to P8 because SHEDS are capable to decrease their proliferative potential and increase the expression of senescence markers (p53, p21 and p16) with higher cell passages in culture [24].

HUVECs were obtained from human umbilical cords and maintained for a maximum of 24 h in magnesium and calcium free phosphate buffered saline (PBSA; Invitrogen) following the methodology previously described [25]. Briefly, the cells were cultured in M199 culture medium (Cultilab, Campinas, SP, Brazil) supplemented with 2 mM L-glutamine (Sigma), 200 μg/mL of Endothelial Cell Growth Supplement (ECGS) (Sigma), 50 μg/mL heparin (Sigma), 20% fetal bovine serum (Cultilab) and 100 IU/mL penicillin, 100 μg/mL streptomycin (Gibco, Life Technologies, Carlsbad, CA, US) and placed in Petri dishes (Corning Inc., New York, NY, US) previously coated with 0.5% gelatin in PBSA. The cells were maintained at 37 °C and the culture medium was changed every 48 hours.

### 2.2 SHEDs conditioned medium (SHED-CM)

To produce the SHED-CM the PDH3 cells were plated in 75 cm^2^ culture flasks (1 × 10^5^ cells/10 mL) in DMEM/Ham's F12 clonogenic medium. Upon confluence, the culture medium was replaced by DMEM/Ham's F12 (Invitrogen) supplemented with 1% antibiotic-antimycotic solution (100 U/mL penicillin, 100 μg/mL streptomycin; Invitrogen). After 3 days, this medium was collected in Falcon tubes and then submitted to centrifugation at 15,000 xg for 5 min. The supernatant (SHED-CM) was transferred to new sterile tubes and stored in freezer at -70 °C.

### 2.3 Cell viability

In order to investigate possible effects of SHED-CM on the proliferation and survival of HUVECs the flow cytometry was used for identifying the markers Ki67 and 7AAD, respectively.

Previously, 6 wells plate were precoated with 0.5% gelatin in PBSA and maintained in an incubator at 37 °C overnight. Then, 5 × 10^5^ HUVECs were plated per well and cultured in three different culture media (3mL/well), according to the experimental groups, as follows: positive control group (M199 medium); negative control group (DMEM/Ham's F12 clonogenic medium) or the experimental medium (SHED-CM medium). The cells were maintained in these culture conditions for 72 h and then submitted to the flow cytometry. Aliquots of HUVECs (1 × 10^6^ cells) of all experimental groups were washed and resuspended in PBS containing conjugated primary monoclonal antibodies (1:200) specific to Ki67 (ABCAM, Cambridge, UK) and 7AAD (Gibco); both used according to the respective manufacturers. A total of 50.000 events were analyzed by flow cytometer (FACS Canto, BD Biosciences) using the 9.6.2 FlowJo Software Version (Tree Star).

### 2.4 Matrigel^®^ angiogenesis assay

In order to induce the formation of 3D vascular-like structures, HUVECs were plated on the top of Matrigel^®^ (BD Biosciences) as previously described [26]. Briefly, Matrigel^®^ was previously thawed overnight (4 °C). Thus, 400 μL of cold Matrigel^®^ was placed to each well (24 wells plate) and maintained at 37 °C in incubator for 1 hour to achieve the gel consistency. Next, 1 × 10^5^ cells resuspended in culture medium according the experimental groups (e.g. M199; DMEM/Ham's F12 or SHED-CM) were plated on the top of the Matrigel^®^. The cells were maintained in an incubator (Baker Ruskinn - Invivo2 300, Sanford, ME, US), at 37 °C in hypoxic conditions (1.5% O_2_, 5% CO_2_ and 93.5% N_2_). After 24 h, images of the cultures of all experimental groups were captured with a phase contrast inverted microscope (Carl Zeiss, Oberkochen, Germany) at the magnification of 40X. To quantify the amount of vascular-like structures 15 phase photomicrographs of each group were analyzed using the NIH Image J Program. Only vascular-like structures without any disruption were considered for counting.

### 2.5 Cell migration

The “scratch” test was used for following the cell migration according to the protocol proposed by Liang et al. [27]. Previously, 100 mm diameter Petri dishes were precoated with 0.5% gelatin and maintained overnight in an incubator at 37 °C. Thus, 1 × 10^5^ HUVECs were plated with 4 ml of M199 complete medium in each well. Three wells were plated for each experimental group. Upon confluence, a scratch was performed in the cell monolayers using a pipet tip (p200), as described elsewhere [28]. Then, the dishes were washed with PBS and fresh media were applied according to the groups: M199 (positive control); DMEM/Ham's F12 medium (negative control) or SHED-CM (experimental medium). Cells in the wounded area were counted after 3h, 6h, 9h, and 12h, with images captured (10 per group at each time) in phase contrast inverted microscope and digital camera at the 40X magnification.

### 2.6 Real-time quantitative reverse transcription-polymerase chain reaction (RT-qPCR)

To observe the effects of SHED-CM on *VEGF*-A gene expression in HUVECs, the RT-qPCR was performed. Each of the three cultures media were applied: M199 (positive control); DMEM/Ham's F12 medium (negative control) or SHED-CM (experimental medium). After 24 h of contact with the media the total mRNA from the cultured HUVECs was extracted using the TRIzol Reagent (Invitrogen) according to the manufacturer's protocol. Briefly, the mRNA concentration and purity were measured with a Nanodrop 2000 spectrophotometer (Thermo Scientific, Rockford, Illinois, US. We used samples with a relative absorbance ratio between 1.8 and 2.0 at 260/280. The quantification of specific mRNAs was conducted using an ABI Prism 7300 Sequence Detection System (Applied Biosystems, Foster City, CA, US) with the SYBR Green real-time PCR assay (Applied Biosystems) for the gene *VEGFa* (Forward) 5′ CCTTGCTGCTCTACCTCCAC 3′, (reverse) 5′ ATGTTGGACTCCTCAGTGGG 3′; gene expression was normalized to PPIA expression (Hs99999904_m1; Taqman real-time PCR assay Applied Biosystem); RT-PCR was performed using the Taqman real-time PCR assay (Applied Biosystem) for the gene *PPIA* (Hs99999904_m1). Cycling conditions were: 10 minutes at 95 °C followed by 40 cycles at 20 seconds each at 95 °C, 20 seconds at 58 °C, and 20 seconds at 72 °C. Analysis used Sequence Detection Software 1.9 (SDS; Applied Biosystems). Gene expression was normalized to PPIA expression.

### 2.7 Enzyme-linked immunosorbent assay (ELISA)

In order to detect and measure the VEGF in the culture media before and after contact with HUVECs, the ELISA assay was done using the ELISA Development Kit (Peprotech, Ribeirão Preto, SP, Brazil). The media were: M199 (positive control); DMEM/Ham's F12 medium (negative control) or SHED-CM (experimental medium) before and after 48h in contact with HUVECs.

The reagents for the ELISA assay were prepared, as follows: 100 μL of solution containing the capture antibody (1 μg/mL) was added to 96-well plates and incubated overnight at room temperature (RT). After this period, the wells were washed and blocked with blocking buffer. Then, 100μL of standard or of each type of culture medium were added and incubated at RT for 2 hours. After washing, 100μL of solution containing the detection antibody (1 μg/mL) was added and incubated at RT for 2 hours and then washed. The plates were incubated with 100μL of a solution containing avidin peroxidase (dilution at 1:1500) at RT for 30 minutes. After another washing, 100μL of substrate solution was added in each well and then incubated at RT for 5 minutes for color development. The detection was performed by an ELISA plate reader Synergy H1 (Biotech, Winnoski, VT, US) at 450 nm, according the manufacturer's instructions.

### 2.8 *In vivo* experiment

The angiogenic effects of the SHED-CM were also analyzed *in vivo*. For this study 10 weeks-old rats (*Rattus norvegicus albinus*) were used in a rodent ortotopic model of dental pulp regeneration, as previously described [29]. All procedures were approved by the Faculdade de Odontologia animal care committee (Protocol nº #1112/2011).

**Fig. 1:**
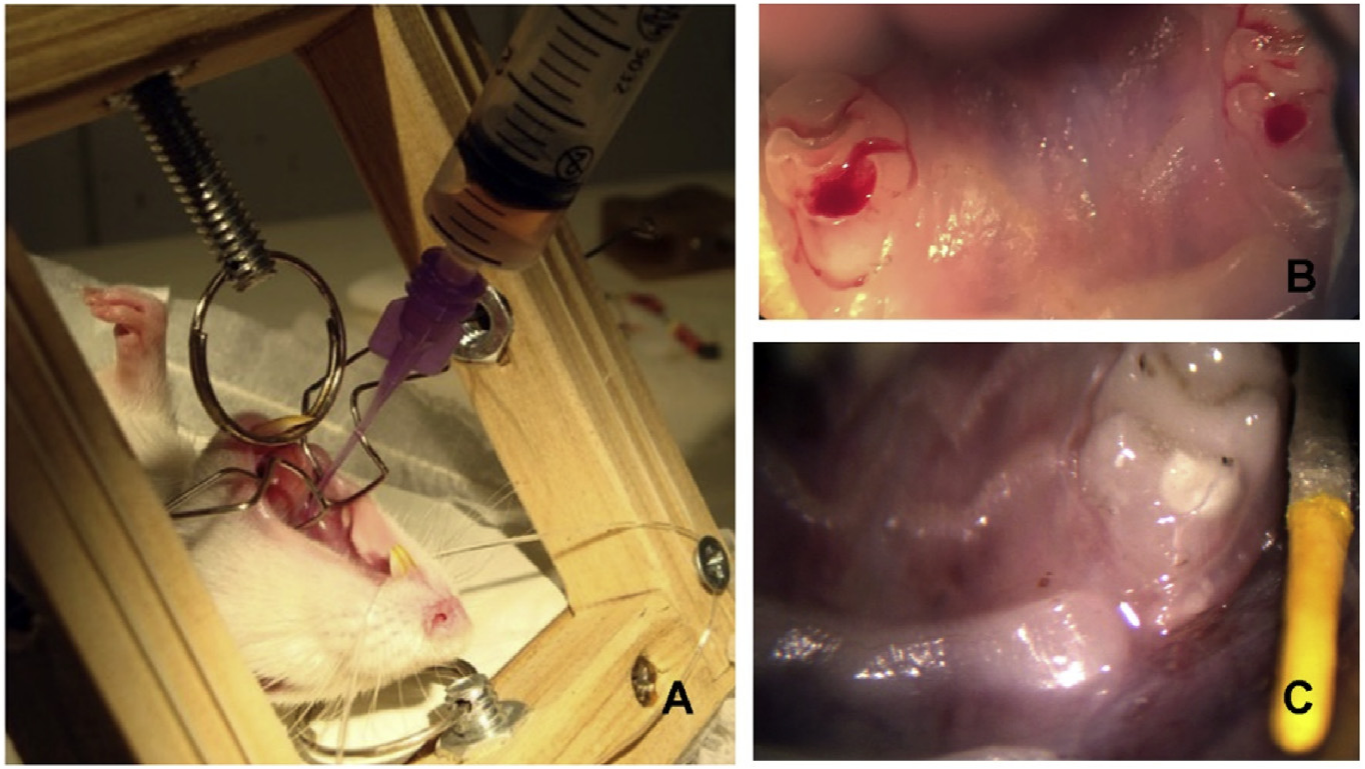
Illustrative photographs of the *in vivo* experimental procedure. (A) Fulfilling the root canal with the SHED-CM; (B) blood clot; (C) restored tooth.

Briefly, rats were anesthetized [ketamine (80–120 mg/kg) and xylazine (5–10 mg/kg), intraperitoneally] and the endodontic access was made at the central surface of the upper first maxillary molar tooth with a handpiece (NSK), and a round carbide bur (#¼ size, Dentsply Caulk, Milford, DE, US), in low speed with portable dental unit (Dentport Dental Supply, New Smyrna Beach, FL, US). The root canal instrumentation was performed using endodontic K-files, been the last instrument the #25 file (Dentsply Maillefer, Ballaigues, VD, Switzerland) associated with irrigation using 1% sodium hypochlorite (NaOCl). After root canal instrumentation, the animals were maintained in 2 different groups: SHED-CM group (n = 3; Fig. 1A), when the root canals were submitted to the effects of the conditioned medium, and the Control Group (n = 2), where the root canal did not receive any treatment. In both groups an ISO 20 K-file was introduced slightly beyond the apex. We instrumented 1 mm beyond the apical foramen to induce bleeding until the entire canal was filled by the blood clot (Fig. 1B). Before the procedure, the CM was thawed and immediately applied in the entrance of the root canal, on the top of blood clot, with the use of an irrigation syringe. Finally, the crown sites of SHED-CM and Control teeth were sealed with coltozol (Coltene, Altstatten, Switzerland) and light cured composite resin (Filtek Z250, 3M ESPE; Quadrant LC, Cavex, Haarlem, Netherland) (Prime and Bond, Dentsply) (Fig. 1C). For the best survival condition the animals were kept at standard temperature (20–24 °C) and sacrificed 30 days later. After sacrifice the maxilla of each animal was dissected and this sample fixed in 10% paraformaldehyde, decalcified with 10% EDTA, stained with H&E and then, examined under a light microscope.

### 2.9 Immunohistochemistry

To evaluate the presence of vessels in the neoformed pulps were performed immunohistochemical experiments [30]. Briefly, formalin-fixed paraffin-embedded tissue sections (3 μm-thick) were applied on previously silane-coated slides. After the deparaffinization, the sections were rehydrated in decrescent ethanol-graded series. We used 3% H_2_O_2_ solution (45 minutes) to block the endogenous peroxidase. Finally, we used Protein Block (X0909, Dako, Carpinteria, CA, US) (10 minutes) to avoid the non-specific binding.

Polyclonal rabbit anti-VEGFR2 (1:700; Sigma-Aldrich) primary antibody was applied over the slices and maintained in a humid chamber (4 °C) overnight. Next, the slices were incubated with the detection system based in Chain Polymer-Conjugated Technology (EnVision^®^, Dako) for 30 minutes at room temperature (RT). Then, the immunostaining was revealed using a chromogenic substrate mixture (3,3′-Diaminobenzidine; D5905, Sigma-Aldrich). Finally, Harry's hematoxylin was used to counterstain the sections. For positive control, we used normal dental pulp from rats while in the negative control we suppressed the primary antibody during the procedures.

### 2.10 Statistical analysis

The results were checked for normality by the Kolmogorov Smirnov or DÁgostino & Pearson test. Proliferation and viability cell data were analyzed using Kruskal-Wallis' test and Dunn's Multiple Comparison as post-hoc test. Data from migration test and ELISA were evaluated using Two-way ANOVA and Bonferroni as post-hoc test. The vascular-like structure formation and data from the RT-qPCR were evaluated using One-way ANOVA and Multiple Comparisons Tukey's test as post-hoc test. Two-tail analyzes were used in all tests, and the different values of p were represented as follows: *p ≤ 0.05; **p ≤ 0.01; ***p ≤ 0.001.

## 3 Results

### 3.1 PDH3 cells expressed mesenchymal stem cell surface markers

**Fig. 2:**
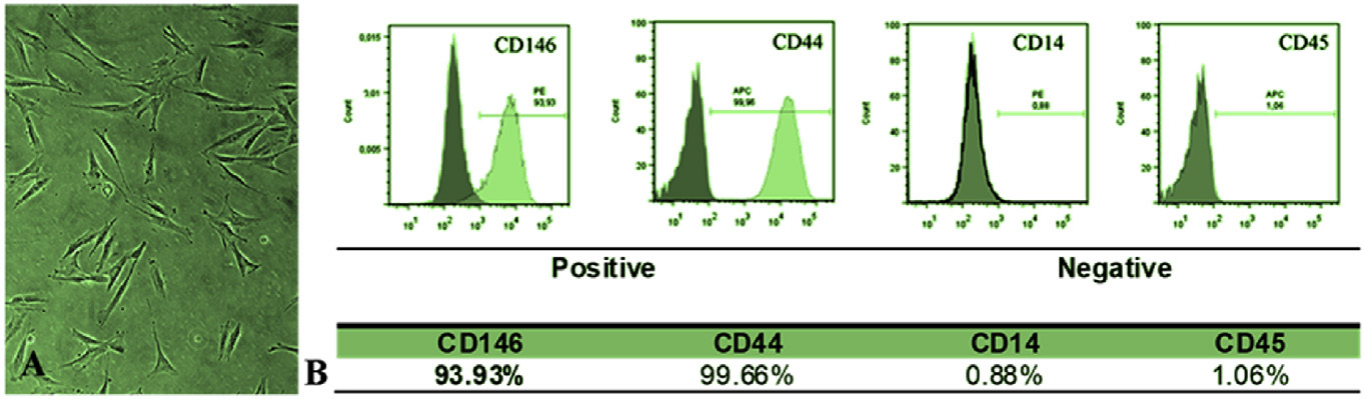
(A) Representative phase photomicrograph of PDH 3 cells and (B) the immunoprofile of the surface molecules. Positive for CD146 and CD44 and negative for CD14 and CD45.

PDH3 cells used to prepare the conditioned media expressed typical levels of mesenchymal stem cell surface markers (Fig. 2). The cultures expressed positivity for MSC markers (CD44 and CD146), whereas the non-specific MSC cell markers (CD14 and CD45) were absent.

### 3.2 SHED CM promoted proliferation and prevented cell death

**Fig. 3:**
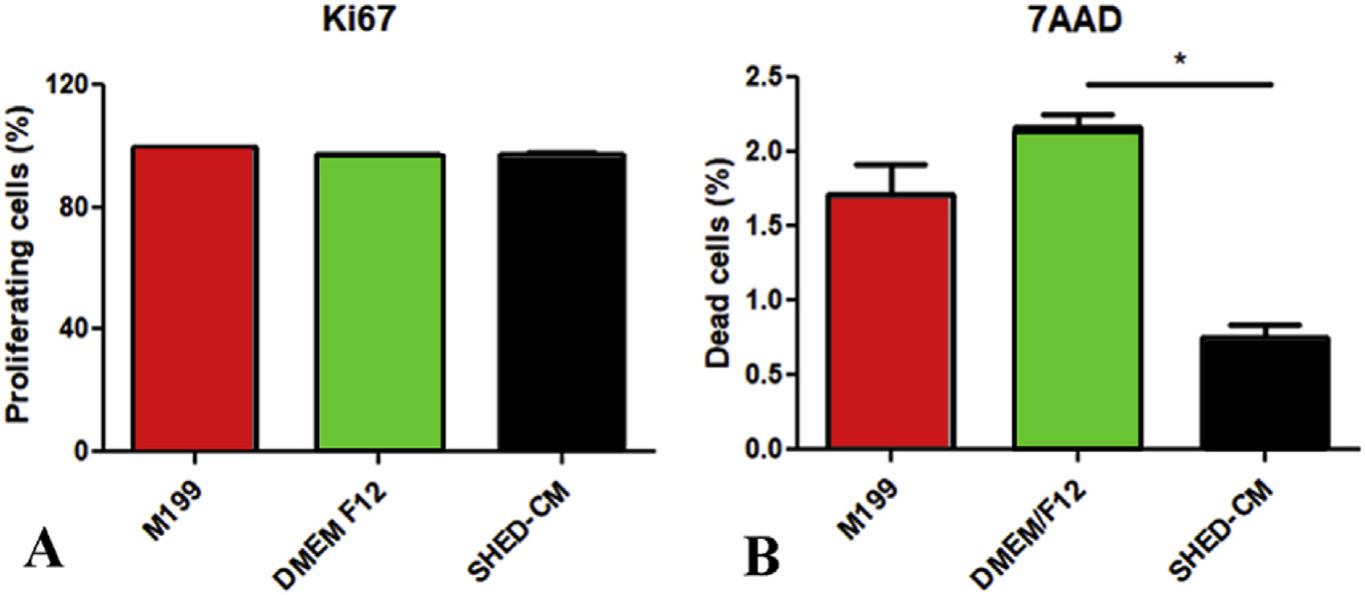
Graphic representation of the percentage of expression of the proliferation marker Ki67 (A) and cell death marker 7AAD (B) in HUVECs after contact with the experimental media. Different letters indicate significant differences (*p < 0.05).

Fig. 3 presents the results of the effects of all tested culture media in the expression of the cell proliferation marker Ki67 (Fig. 3A) and the cell death marker 7AAD (Fig. 3B). The three media tested stimulated cell proliferation. Positive expression of Ki67 was observed in a similar amount in all groups. Additionally, SHED-CM exhibited the smallest amounts of cell death marker (7AAD) (p < 0.01).

### 3.3 SHED conditioned medium promoted vascular-like structures formation

**Fig. 4:**
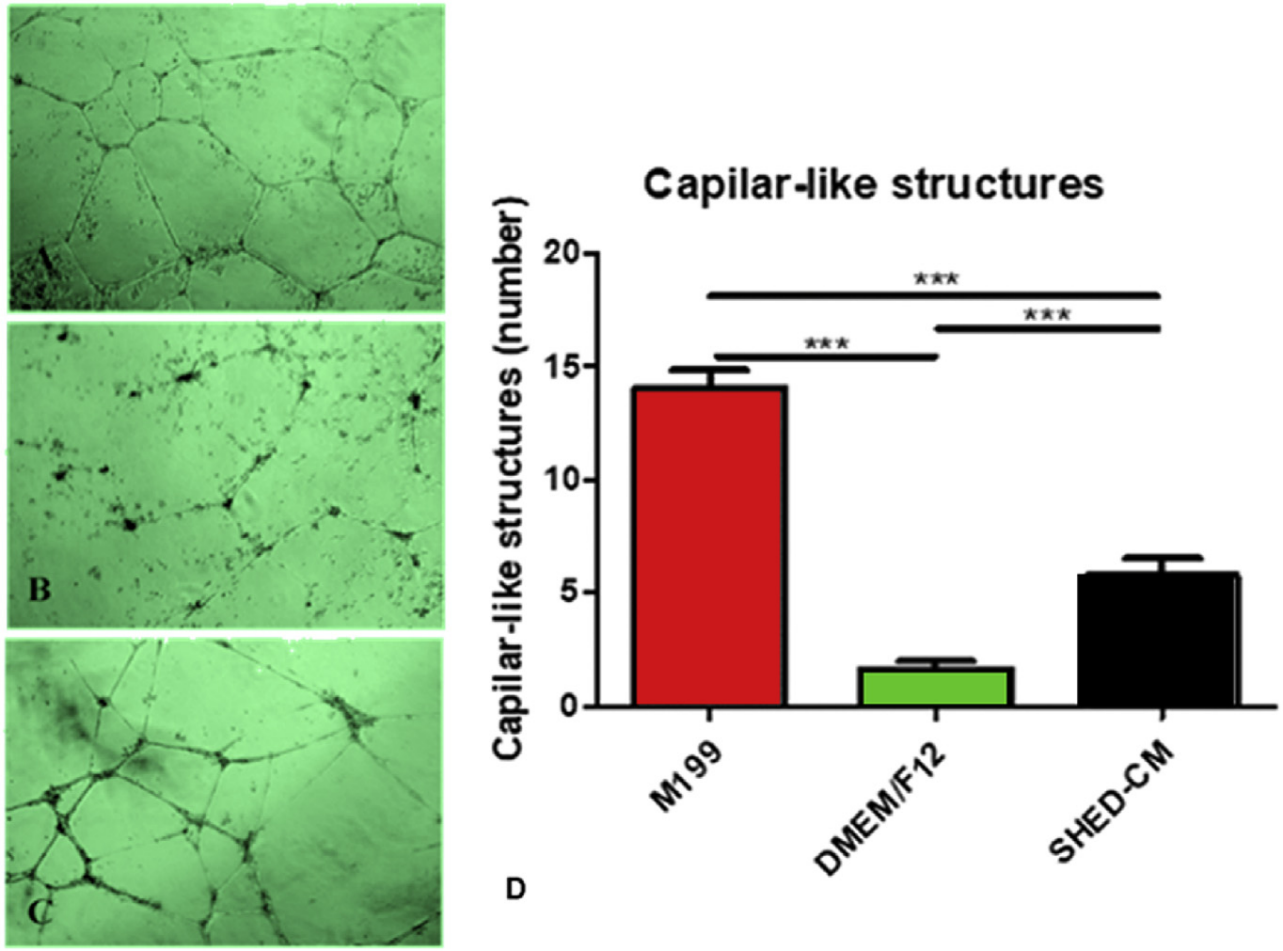
Representative phase photomicrographs of vascular-like structures of HUVECs grown on Matrigel after contact with the three different media: (A) M199; (B) DMEM/Ham's F12 and (C) SHED-CM; and (D) graphic representation of the amount of these structures [# M199 is different from the DMEM/Ham's F12 (p < 0.0001) and from SHED-CM (p = 0.0421); *DMEM/Ham's F12 is different from SHED-CM (p < 0.0043)].

Vascular-like structures were observed in all groups (Fig. 4). The HUVECs plated on the top of Matrigel^®^ and maintained in hypoxia under the influence of all media during 24 h were able to form vascular-like structures with significant differences in quality (Fig. 4A–C) and amounts (Fig. 4D). As expected, the highest amount of vascular-like structures was observed in the positive control group (M199) and the smallest in the negative control (DMEM/Ham's F12), whereas the SHED-CM group presented an intermediate number of these structures (Fig. 4D).

### 3.4 Conditioned medium promoted cell migration

**Fig. 5:**
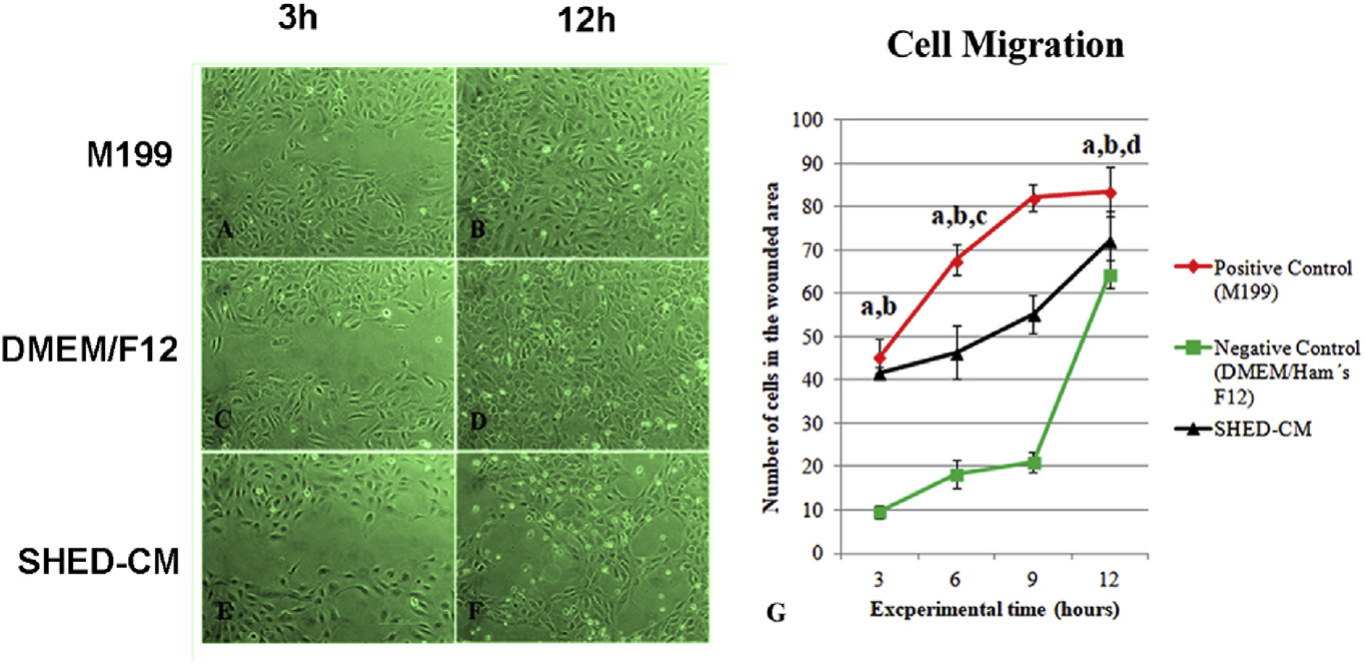
Representative phase photomicrographs of HUVECs in the scratch assay (A–F). Graphic representation of the number of cells inside the wound of HUVECs monolayers after contact with the all media (*a* p < 0,001 M199 versus DMEM/Ham's F12; *b* p < 0,001 SHED-CM versus DMEM/Ham's F12; *c* p < 0,01 M199 versus SHED-CM; *d* p < 0,001 M199 versus SHED-CM).

The behavior of HUVECs related to migration was similar to that of vascular-like structures formation. In fact, in the SHED-CM group an intermediated migration speed was observed (Fig. 5). Until 12 h the faster wound closure process occurred in the positive control group (M199) and the worst in the negative control group (DMEM/Ham's F12) (p < 0.001) (Fig. 5A, C, E). During migration the cells maintained their original morphology of a slightly rounded polygonal shape. At 36 h all experimental wounds were closed, except those maintained in SHED-CM, where a cellular morphological modification was observed (Fig. 5B, D, F). In this group, cells became stretched and organized in a honeycomb aspect throughout the wound area (Fig. 5F).

### 3.5 SHED conditioned medium promoted *VEGFa* expression

**Fig. 6:**
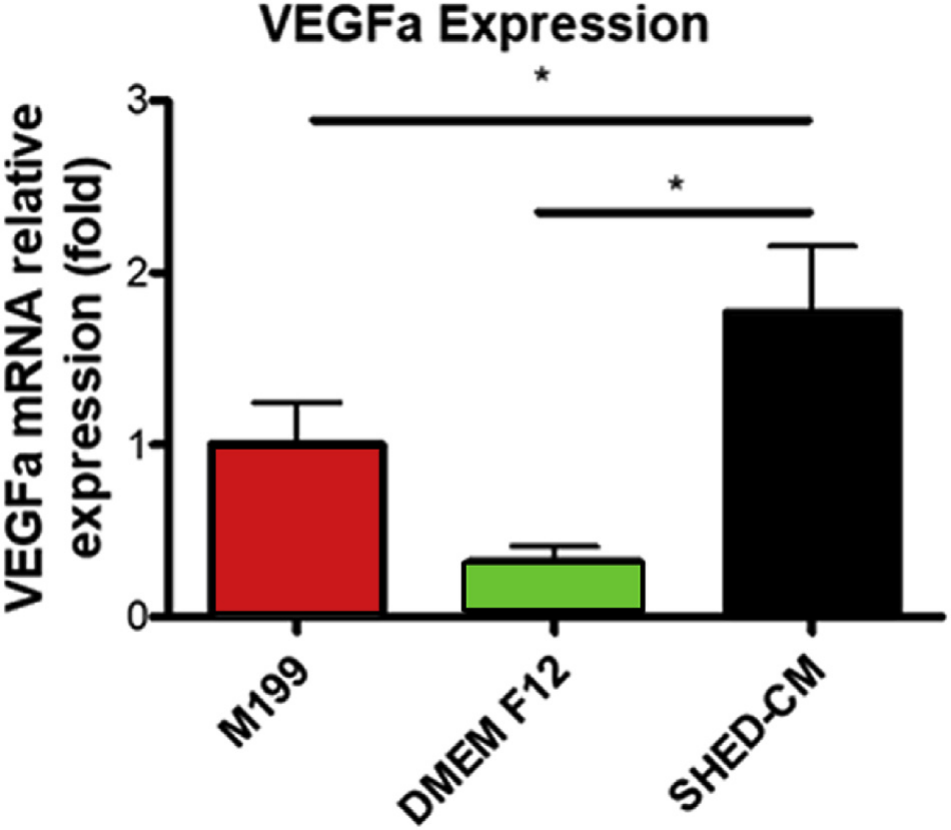
Graphic representation of the relative expression of *VEGFa* in HUVECs after contact with the media. (*p < 0.001).

Positive expression of *VEGFa* was observed in all groups (Fig. 6). The SHED-CM group presented an expression of *VEGFa* similar to that of positive control group (M199) and significantly higher (p < 0.05) when compared with the negative control group (DMEM/Ham's F12).

### 3.6 SHED conditioned medium increased VEGF synthesis in culture

**Fig. 7:**
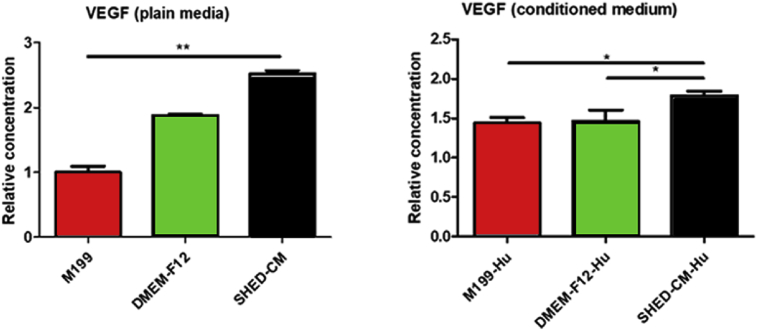
Graphic representation of the amount of VEGF in the media prior to contact with HUVECs (w/o cells) and after 48h of HUVEC contact (HUVEC). The values were calculated relative to M199 medium (before HUVEC contact). (*p < 0.05; **p < 0.01).

The comparison of the concentration of VEGF in the tested culture media prior to the contact with the HUVECs (plain media) showed that SHED-CM presented the highest concentration (p < 0.01), and the positive control medium (M199) the smallest (p < 0.01; Fig. 7).

The media conditioned by HUVECs grown in M199 presented significant increase in concentration when compared with the plain M199 medium (p < 0.01). The media conditioned by HUVECs grown in the negative control medium (DMEM/Ham's F12) and the SHED-CM medium presented decrease in the concentration of VEGF when compared with the correspondent plain medium. The SHED-CM conditioned by the HUVECs presented higher concentration of VEGF than all other HUVECs'conditioned media (p < 0.01).

### 3.7 SHED conditioned medium induced tissue formation inside the root canal

**Fig. 8:**
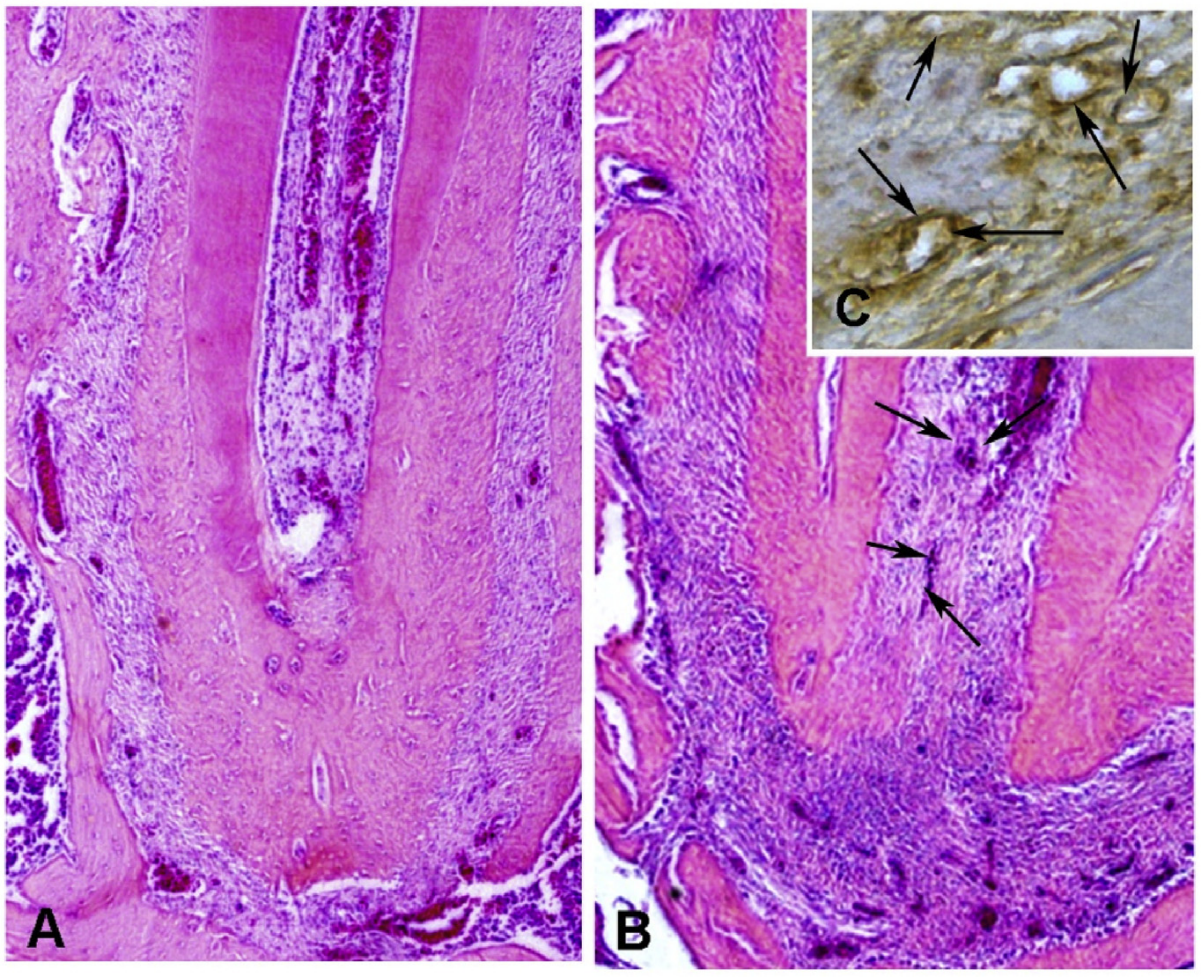
Representative photomicrographs of normal dental pulp of rats (A) and the newly connective tissue similar to dental pulp inside the apical area of the root canal (B, C). The newly formed tissue with the blood vessels (between arrows) shows positive reaction to the VEGFR2 antibody (C). (A, B: H&E, original magnification of 100X; C: immunohistochemistry, 400X).

Thirty days after the treatment the rats of the SHED-CM group presented formation of connective tissue inside the root canals, and the teeth of control groups were always with empty root canals. Fig. 8 illustrates the tissue formed inside the root canal induced by the SHED-CM (Fig. 8B, C). The newly connective tissue was occupying an apical area of the root canal space. In the tissue was possible to observe scattered blood vessels, most of them filled with hemaceae, inflammatory cells and collagen fibers. This tissue resembles the dental pulp of the rats (Fig. 8A). The immunohistochemistry reaction against VEGFR2 was strong positive in the blood vessels walls (Fig. 8C).

## 4 Discussion

Neovascularization is of extreme importance in tissue engineering because it brings the necessary nutrients to the migrating cells in the new tissue or organ [31]. The neovascularization process or angiogenesis is the development of blood vessels from pre-existing vascularization. This process involves vasodilatation, increased vascular permeability, plasma protein extravasations, cell migration, proliferation, and new cell migration to distant sites [32]. In the tissue engineering, the formation of new tissues is dependent on stem cells, scaffolds and growth factors. It is known that stem cells release paracrine factors involved in the tissue regeneration process [33, 34]. Thus, the conditioned medium (CM) by stem cells could be an option to the use of stem cells in tissue engineering, once it would minimize the risk of cell rejection after the transplant. As will be described further below, many experiments have showed the potential of CM of stem cells for several applications. In particular, some experiments showed that SHED-CM was more efficient in the production of VEGF than media conditioned by other types of stem cells [35, 36]. Then, with the hypothesis that SHED-CM would release angiogenic factors; the present study analyzed this medium *in vitro* and *in vivo*. We have shown that SHED-CM promoted proliferation, vascular-like structures formation, cell migration, VEGFa expression, VEGF synthesis, impaired cell death and induced tissue formation inside the root canal.

The first experiment of the present study analyzed the effect of different culture culture media (M199, DMEM/Ham's F12 and the SHED-CM) on proliferation (Ki67) and cell death (7AAD) markers in HUVECs. All the three media tested stimulated cell proliferation. On the other hand, the cell death marker (7AAD) was significantly smaller in the cells grown in SHED-CM than in the other groups. It means that the factors released by SHEDs maintained cell proliferation and protect the HUVECs from cell death. Ahmed et al. [37] analyzed the antiapoptotic effect of secretome from DPSC on SH-SY5Y cells exposed to amyloid beta peptide. The secretome stimulating the endogenous survival factor Bcl-2 and decreasing the apoptotic regulator Bax, revealing the possible mechanism of cell protection. Matsushita et al. [36] applied the CM via the jugular region in rats and after 24h observed by immunohistochemistry that the SHED-CM in relation to the BM-CM suppresses the pro-inflammatory factors (TNF-alpha, IL-1Beta and IL-6), increased expression of anti-inflammatory factors (IL-10 and TGF-beta 1) and increased expression of anti-apoptotic factors (SCF and IGF-1). In addition, Mita et al. [35] observed that SHED-CM was capable to improve the cognitive function in mice with induced Alzeimer's disease. Along with that Shimojima et al. [38] observed the immunomodulatory effect of SHED-CM in experimental treatment of autoimmune encephalomyelitis (EAE) in mouse model. Mead et al. [39] observed that DPSC-CM also has neuroprotective and neuritogenic effect, by expression of NGF, BDNF and NT-3. Thus, the SHED-CM showed protective property that would have positive effects on tissue engineering by associating proliferation and cell death impairing.

Besides vascular cell proliferation, other important effect of SHED-CM on tissue engineering would be the ability to induce the formation of vascular structures. Thus, using the hypoxia (1.5% O_2_) culture condition of HUVECs grown on Matrigel under the influence of the three media, this ability was tested. In fact, although the vascular-like structures of the HUVECs grown in the M199 (that is the appropriate medium for this cell type) were observed in higher amount and quality, the SHED-CM was able to induce the formation of these structures in higher amounts than the negative control. In addition to these results, migration is an important process of vascular structures formation. The results of HUVEC migration were similar to that of vascular-like structures formation. This means that factors released by SHEDs improve the formation of vascular-like structures. Gorin et al. [40] cultivated HUVECs in a 3D collagen culture in the presence of SHED-CM and verified 3D network of capillaries formation, with expression of β-catenin, CD31 and podocalyxin. The authors identified that the secretion of VEGF by SHED was proportional to the presence of FGF-2 and that angiogenic potential and secretion of VEGF by SHED were amplified under hypoxia condition. In addition, the inhibition of VEGF affected SHED-induced capillary formation. The same was demonstrated by Lee et al. [41] where the use of VEGF neutralizing antibody hindered tubular formation of HUVECs incubated with DPSC-CM. Our data showed the ability of CM secreted by SHED to induce the formation of vascular structures. As described in the literature, CM of stem cells contained some additional factors that promoted angiogenesis. Thus, we suggest that SHED-CM releases differentiation factors that increase migration capacity of HUVEC thereby contributing to the vascular-like structures arrangement observed on Matrigel culture.

M199 medium is usually poor in nutrients containing Earle's salts, L-glutamine, and sodium bicarbonate, heparin, fetal bovine serum and antibiotics, but is supplemented with ECGS–Endothelial Cell Growth Supplement that contains appropriate factors for endothelial cell culture [42]. DMEM/Ham's F12 that is the appropriate medium for SHEDs culture is rich in fetal serum and several factors such as inorganic salts, aminoacids, and vitamins supplemented with fetal bovine serum, antibiotics, L-glutamine and non-essential amino acids (Sigma). However, these components were not sufficient for maintaining the quality of HUVECs. The protein composition of stem cells secreted factors, as the hMSC-CM, was performed using the cytokine array analysis [36, 43]. The authors observed the presence of at least 48 proteins, including osteoprotegerin, angiogenin, MCP-1, MCP-3, HFG, IL-6 and VEGF-A, commonly found in cell proliferation, cell recruitment and angiogenesis properties. Lee at al [41]. verified mRNA expressions for the angiogenic genes, including VEGF, FGF-2, VEGFR-1, VEGFR-2, PECAM-1, and VE-cadherin and the presence of VEGF and VEGFR1 proteins. Ahmed et al. [37] showed that DPSC contains higher concentrations of VEGF, RANTES, FRACTALKINE, FLT-3, GM-CSF and MCP-1 than factors from BMSC and ADSC. Also, Matsushita et al. [36] observed that levels of proteins in the SHED-CM were more than 1.5–fold greater than that observed in DMEM. VEGF has many functions on angiogenesis. Gifford et al. [44] demonstrated that HUVEC proliferation by VEGF rise in ERK 2 activation, which is associated with mitogens. Esser et al. [45] showed exogenous VEGF applied on HUVEC stimulated cell migration and sprouting with the formation of actin filaments. The latter was associated with tyrosine phosphorylation levels of junction components; this result was accompanied with high amounts of VEGFR-2 and its specific phosphorylation, as well as stimulated the tyrosine phosphorylation of multiple proteins in HUVEC. Between these proteins VEGF induced the tyrosine phosphorylation transmembrane cell-cell adhesion systems like: PECAM-1; and VE-cadherin as well as the associated proteins, which were identified as β-catenin, plakoglobin and armadillo protein p120. The VE-cadherin staining appeared to be codistributed with F-actin rich filopodia. In contrast, Abraham et al. [46] demonstrated VE-cadherin act in a positive feedback loop by antagonizing VEGFR2 necessary for ensuring stable cell-cell junctions. Our results are corroborated by the cited literature. The present work showed that the SHED-CM is rich in VEGF, presented in higher concentration than that of all other media tested. As discussed above, VEGF is the main and common factor among other factors presented in stem cells CM, which brings the characteristic of promoting angiogenesis.

The most striking finding of the present study was the ability of SHED-CM in the formation of a vascularized connective tissue in the root canal of rats. We have already in a previous study analyzed the dental pulp regeneration and the formation of tissue inside the root canal, which was only observed when the tooth was stimulated by photobiomodulation therapy (PBMT). PBMT is able to increase the synthesis of VEGF [47]. Thus, we can suggest that the success in the tissue formation inside the root canal in the present study was due to the action of VEGF content of the SHED-CM. Gorin et al. [40] verified that SHED induces angiogenesis in a structure composed by tooth slices which empty pulp chamber space was filled with type I collagen containing SHED, implanted subcutaneously in mouse back. This *in vivo* simulation of angiogenesis was increased by tissue constructs primed with FGF-2 or hypoxia. Yadlapati et al. [48] developed a polydioxanone fiber, loaded with VEGF, which was used to fill root fragments from human premolars. This structure was implanted subcutaneously in mice to simulate a clinical scenario of endodontic regeneration procedures. The authors observed that the scaffold was biocompatible which presented a negligible number of inflammatory cells. Presence of cells lining the dentinal wall, new blood vessels and connective tissue formation into the root canal space was observed after 45 days of experiment. Our experiment was performed with SHED-CM using rat ortotopic design of dental pulp reposition by insertion a file through apex and apical foramen promoting bleeding and blood clot into root canal. The rats treated with SHED-CM presented formation of connective tissue and was possible to observe scattered blood vessels, most of them filled with hemaceae, inflammatory cells and collagen fibers. This data showed that SHED-CM was able to conduct the slight root restructuring of the dental canal. An immunopositive reaction for VEGFR2 was observed in neoformed tissue, which correlated to the vessels' stages of development.

Here we showed that SHED-CM was able to promote angiogenesis *in vitro* and *in vivo*. It reduced apoptosis while increased migration and vascular-like structures formation *in vitro*. These results were followed by the presence of VEGF in SHED-CM. *In vitro* the role of SHED-CM was intermediary between groups tested, but *in vivo* this CM demonstrated efficient functional property leading to formation of connective tissue similar to dental pulp inside the root canal.

## 5 Conclusion

Here we showed that SHED-CM was able to promote angiogenesis *in vitro* and *in vivo*. It reduced apoptosis while increased migration and vascular-like structures formation *in vitro*. These results were followed by the presence of VEGF in SHED-CM. *In vitro* the role of SHED-CM was intermediary between groups tested, but *in vivo* this CM demonstrated efficient functional property leading to formation of connective tissue similar to dental pulp inside the root canal. This way, together with perspective of low immune rejection, stem cells conditioned medium could be useful for reparative treatments; at least, SHED-CM could be interesting for endodontics reparative treatments.

## Declarations

### Author contribution statement

Sueli Patricia Harumi Miyagi de Cara, Fernando de Sá Silva: Performed the experiments; Contributed reagents, materials, analysis tools or data; Wrote the paper.

Clarice Silvia Taemi Origassa, Danilo Candido de Almeida, Ana Clara Fagundes Pedroni, Giovanna Lopes Carvalho: Performed the experiments.

Maria Stella N. A. Moreira: Performed the experiments; Wrote the paper.

Diego Pulzatto Cury: Analyzed and interpreted the data.

Niels Olsen Saraiva Câmara: Conceived and designed the experiments.

Márcia Martins Marques: Conceived and designed the experiments; Analyzed and interpreted the data; Contributed reagents, materials, analysis tools or data; Wrote the paper.

### Funding statement

This research did not receive any specific grant from funding agencies in the public, commercial, or not-for-profit sectors.

### Competing interest statement

The authors declare no conflict of interest.

### Additional information

No additional information is available for this paper.
